# The I′-chart: a preliminary investigation of an improved individuals chart for quality improvement and control

**DOI:** 10.1093/intqhc/mzag159

**Published:** 2026-09-26

**Authors:** Jacob Anhøj, Wayne A Taylor, Mohammed A Mohammed

**Affiliations:** Centre of Diagnostic Investigation, Rigshospitalet, University of Copenhagen, Copenhagen, Denmark; Taylor Enterprises, North Barrington, Illinois, United States; Faculty of Health Studies, University of Bradford, Bradford, England, United Kingdom; The Strategy Unit, NHS Midlands and Lancashire, Midlands, Birmingham, United Kingdom; Cardiff Business School, The University of Cardiff, Cardiff, Wales, United Kingdom

**Keywords:** Statistical process control, I-chart, Improved I-chart, I'-chart, I prime chart, Variable subgroup size

## Abstract

**Background:**

Statistical process control (SPC) charts are widely used in healthcare to monitor and improve quality. Selecting the appropriate chart can be challenging because healthcare data often comprise subgroup summary measures derived from varying numbers of observations. The traditional Individuals (I-) chart is popular because of its simplicity but assumes constant subgroup sizes, an assumption frequently violated in practice. This can lead to inappropriate control limits and misleading interpretation. We describe the I′-chart, an extension of the traditional I-chart that accommodates varying subgroup sizes while remaining applicable to both measurement and attribute data, and present a preliminary evaluation of its performance.

**Methods:**

The I′-chart was evaluated using a range of simulated and real-world healthcare datasets to address three practical questions: (i) How does the I′-chart compare with the traditional I-chart when subgroup sizes are constant? (ii) How does the I′-chart accommodate varying subgroup sizes? (iii) How does the I′-chart compare with other established SPC charts?

**Results:**

(i) The I′-chart produced identical results to the traditional I-chart when subgroup sizes were constant, confirming that it generalizes the conventional I-chart. (ii) When subgroup sizes varied, the I′-chart adjusted the control limits according to subgroup size, making differences in data precision explicit and, in a healthcare example, changing an apparent special-cause signal into expected common-cause variation. (iii) The control limits produced by the I′-chart using simulated data were broadly comparable with those from established SPC charts, including the Xbar-chart for measurement data and Laney’s P′- and U′-charts for attribute data.

**Conclusion:**

The I′-chart has superior design properties to the traditional I-chart while retaining its simplicity and ease of interpretation. By explicitly accounting for varying subgroup sizes, it addresses an important limitation of the traditional I-chart. Our findings support replacing the traditional I-chart whenever subgroup summary data with varying subgroup sizes are analysed. Although further methodological and empirical evaluation is required to establish its operating characteristics and define its role alongside other SPC charts, the examples presented suggest that the I′-chart produces results comparable with established SPC methods while providing a single, generalizable framework for analysing subgroup summary data.

## Introduction

Statistical process control (SPC) is widely used in healthcare to monitor and improve the quality and safety of care delivery processes. At its core, SPC methodology distinguishes between two types of variation: common cause variation, which reflects inherent fluctuations within a stable process, and special cause variation, which signals a change in the underlying process due to an assignable cause [1–5].

SPC charts are line graphs that help users visualize the behaviour of data from a process over time and identify signals of special cause variation using statistically defined control limits.

There are scores of SPC charts, and choosing the appropriate chart can be less than straightforward, especially in healthcare, where data structures are often complex and varied. In practice, diverse data types such as measurements, counts, proportions, and rates with varying denominators are common—each requiring a different type of control chart depending on assumptions about the underlying statistical distribution of the data, which may be difficult or impossible to verify in practice [6, 7].

Compounding this challenge is the fact that some practitioners lack formal statistical training, and software defaults often promote the use of a single chart type, typically the Individuals I-chart, regardless of whether it is the most appropriate. These issues can lead to the misapplication of SPC charts, generating misleading signals and undermining improvement efforts.

Among SPC charts, the I-chart (also known as the XmR chart) is especially popular for its simplicity and flexibility. It was originally developed for settings where data are collected as single measurements over time—for example, daily blood pressure readings or individual lab test turnaround times—but has proven useful also for attribute data like complication rates or procedure compliance. For this reason, the I-chart is often referred to as the ‘Swiss Army Knife’ of SPC [8].

Unlike other SPC charts, the I-chart estimates process variation directly from the moving ranges between successive data points [9]. So the I-chart operates under a key assumption: that each data point reflects the same underlying area of opportunity—for example, comparable patient volumes, observation periods, or sample sizes.

In many healthcare contexts, this assumption does not hold. For instance, the number of patients with post-surgical complications depends on the number of patients who underwent surgery—in practice, this denominator subgroup size varies over time. When such variation in subgroup sizes exists, the use of the I-chart becomes problematic, as it assumes a constant area of opportunity. This mismatch can result in misleading control limits, which is why the use of the I-chart for such data remains controversial.

To address this limitation, Taylor developed the normalized I-chart [10, 11], a modified version of the traditional I-chart designed to accommodate varying subgroup sizes. Throughout this paper, we refer to Taylor’s normalized I-chart as the I′-chart (I prime chart). Although the method has been described previously by Taylor, it has not been evaluated in a peer-reviewed healthcare journal. We therefore present the theoretical basis of the I′-chart together with a preliminary evaluation using simulated and real-world healthcare datasets, placing the method within the wider SPC literature for healthcare improvement. Our paper is intended as an introductory and illustrative contribution for a healthcare improvement audience, focusing on dissemination and practical application rather than comprehensive comparison with the full family of SPC charts. Accordingly, the examples and simulations are designed to support understanding and practical use, with more extensive methodological evaluation identified as an important area for future research. Our study is guided by the following practical questions:

- How does the I′-chart compare to the I-chart for constant subgroup sizes (of *n* = 1)?
- How does the I′-chart accommodate varying subgroup sizes?
- How does the I′-chart compare to other widely used SPC charts?

## Materials and methods

### Procedure for calculating centre line and control limits

The general formula for calculating control limits for SPC [12] charts is:


control limits=CL±3σ

where *CL* is the centre line (usually the process mean) and *σ* (sigma) is the estimate of process variation used to calculate the control limits. Although the general form of the control limits is common across traditional SPC charts, the method used to estimate σ differs according to the type of data and the particular control chart being used.

SPC charts such as the I-chart are designed for data with a constant subgroup size, most simply where each plotted value is a single observation. Here, σ represents the standard deviation of the underlying process, estimated from the moving ranges between successive data points, since subgroup size is assumed to be constant and so plays no role in the calculations.

SPC charts that are designed to plot subgroup summary statistics (e.g. means or proportions) also estimate σ as the standard deviation of the underlying process, but the control limits are calculated using the standard error of the summary statistic, which depends on subgroup size. The I-chart, however, has no such mechanism.

In particular, *σ* for the I-chart is calculated using:


$$\sigma =\frac{\bar{MR}}{1.128}$$

where $\bar{MR}$ is the average moving range, that is the average of the absolute differences between successive data points.

The control limits are then calculated using:


$$CL\pm 2.66\bar{MR}$$

The traditional I-chart estimates sigma from the moving ranges between successive individual observations and is therefore appropriate when each plotted point represents a single observation or when subgroup sizes are constant. Conventional SPC addresses varying subgroup sizes by selecting different control charts (e.g. $\bar{\text{X}}$–S, P, and U charts), each of which incorporates subgroup size using its own method for estimating sigma [6, 10, 13]. The I′-chart provides an alternative, generalizable framework by estimating sigma from the moving ranges of successive subgroup summaries, standardized for subgroup size, while allowing the control limits to vary according to subgroup size, thereby extending the traditional I-chart to subgroup summary data with varying subgroup sizes [10, 11]. The I′-chart formulae are shown below, using the following symbols:

- *X_i_* = numerator for the *i*-th subgroups
- *n_i_* = subgroups (denominator) of *i*-th subgroups
- *N_i_* = number of subgroups
- *i* = subgroup index (1 to *N*)

Values to plot:


$$y_{i}=\frac{x_{i}}{n_{i}}$$

Centre line:


$$CL=\frac{\sum x_{i}}{\sum n_{i}}$$

Sigma of the ith moving range:


$$\sigma_{i}=\sqrt{\frac{\pi }{2}}\frac{|y_{i}-y_{i-1}|}{\sqrt{\frac{1}{n_{i}}+\frac{1}{n_{i-1}}}}$$

Average sigma:


$$\overset{‾}{\sigma }=\frac{\sum \sigma_{i}}{N-1}$$

Control limits:


$$C\text{ontrol}\;\text{limits}\;\text{for}\;i\text{th}\;\text{data}\;\text{point}=CL\pm 3\frac{\overset{‾}{\sigma }}{\sqrt{n_{i}}}$$

Note that when all subgroup sizes ($n_{i}$) all equal, the above formula reduces to the traditional I-chart form: $CL\pm 2.66\bar{MR}$.

### Data and software

To demonstrate the application of I′-charts on measurement data with varying subgroup sizes, we used data from a regional outpatient clinic for children with diabetes. For privacy purposes, data were aggregated in advance, including the monthly number of children who visited the clinic and their average glycated haemoglobin (HbA1c, a measure of long-term blood glucose levels).

To demonstrate the application of I′-charts on attribute data, we used data on bacteraemia cases and mortality.

To construct the control charts, we used R version 4.5.0 [13] with the add-on package qicharts2 version 0.8.0 [14], which contains functions for traditional Shewhart control charts in addition to functions for Laney’s prime charts (P′ and U′) [15] and now includes the I′-chart. As with the original I-chart, qicharts2 adopts Nelsons’ screening procedure [9], which removes moving ranges greater than the theoretical upper natural limit (=3.2665 × average sigma) before calculating the process control limits. Taylor uses a median range version of the I′-chart to address the same issue [10, 11].

Additionally, we constructed many I′-charts using both pseudo-random data from normal, binomial, and Poisson distributions as well as real-world data sets from healthcare. The pseudo-random data were generated to mimic common cause variation, exclude special cause variation, and use subgroup sizes restricted to a narrow range, supporting illustrative comparison rather than formal performance assessment. The results should therefore be interpreted as indicative rather than definitive prompting further study.

Additional figures, data tables and R code to reproduce the figures are available in the supplementary files (iprime_supp_figs.pdf and iprime_supp_code.pdf).

## Results

The following plots highlight our main findings based on real-world or simulated data.

### I′-chart for measurement data

Figure 1 shows an I-chart of monthly HbA1c averages from children with diabetes. As expected, the control limits exactly match those of the corresponding I′-chart with denominators all set to 1 (not shown). Notice the data point above the upper control limit in April 2020 suggesting a special cause. However, when plotting aggregated measurement data, the I-chart does not account for variations in subgroup size.

**Figure 1:**
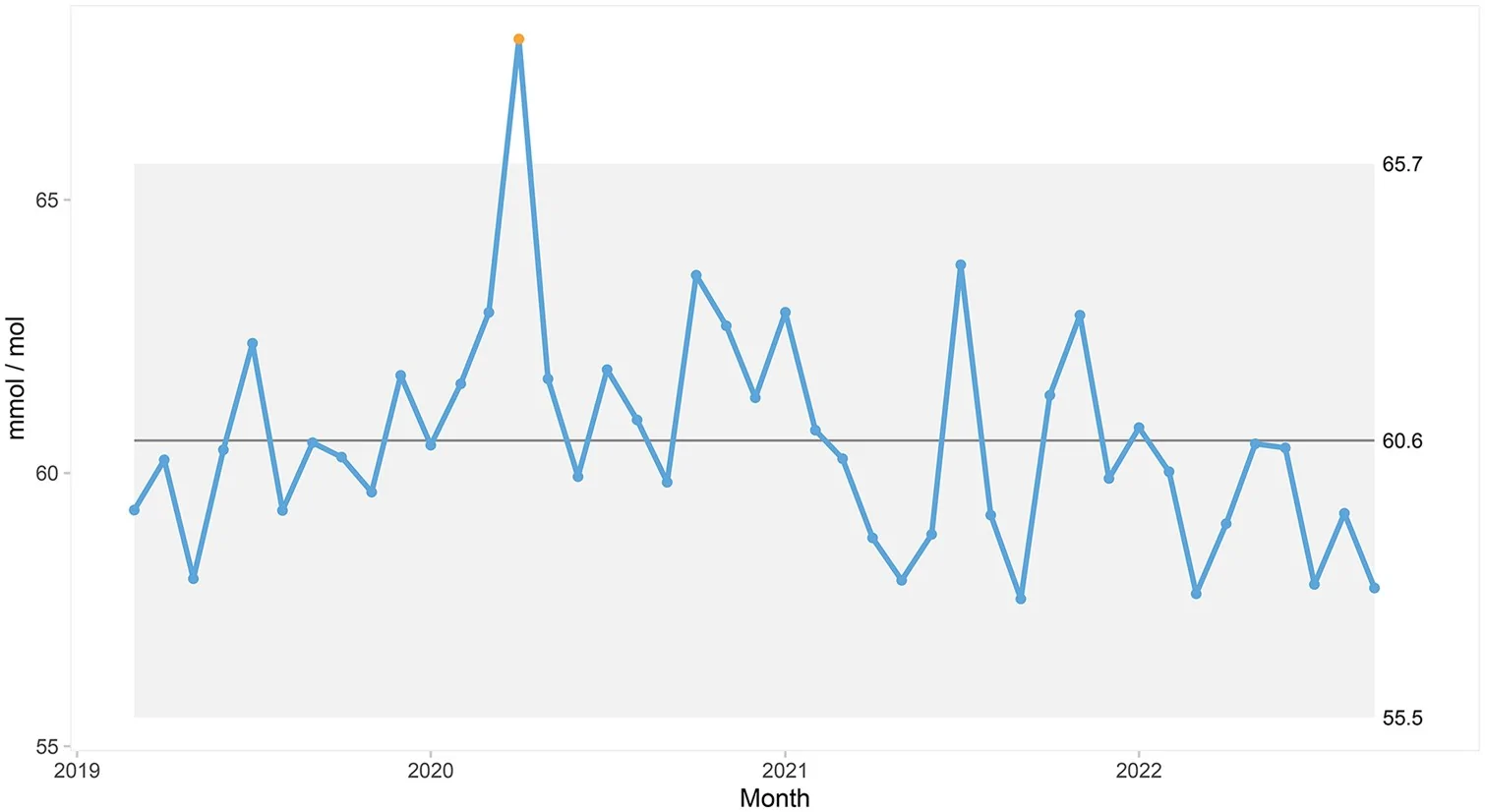
I-chart of average HbA1c without denominator. Shaded area represents the area between the control limits.

The I′-chart in Fig. 2 takes the subgroup size (number of patients) into account and adjusts the control limits correspondingly. April 2020 was the first month of lockdown during Covid-19 in Denmark, and the number of patients seen during this month was significantly lower than usual, which allowed for larger-than-usual common cause variation in measurements and consequently wider control limits this month.

**Figure 2:**
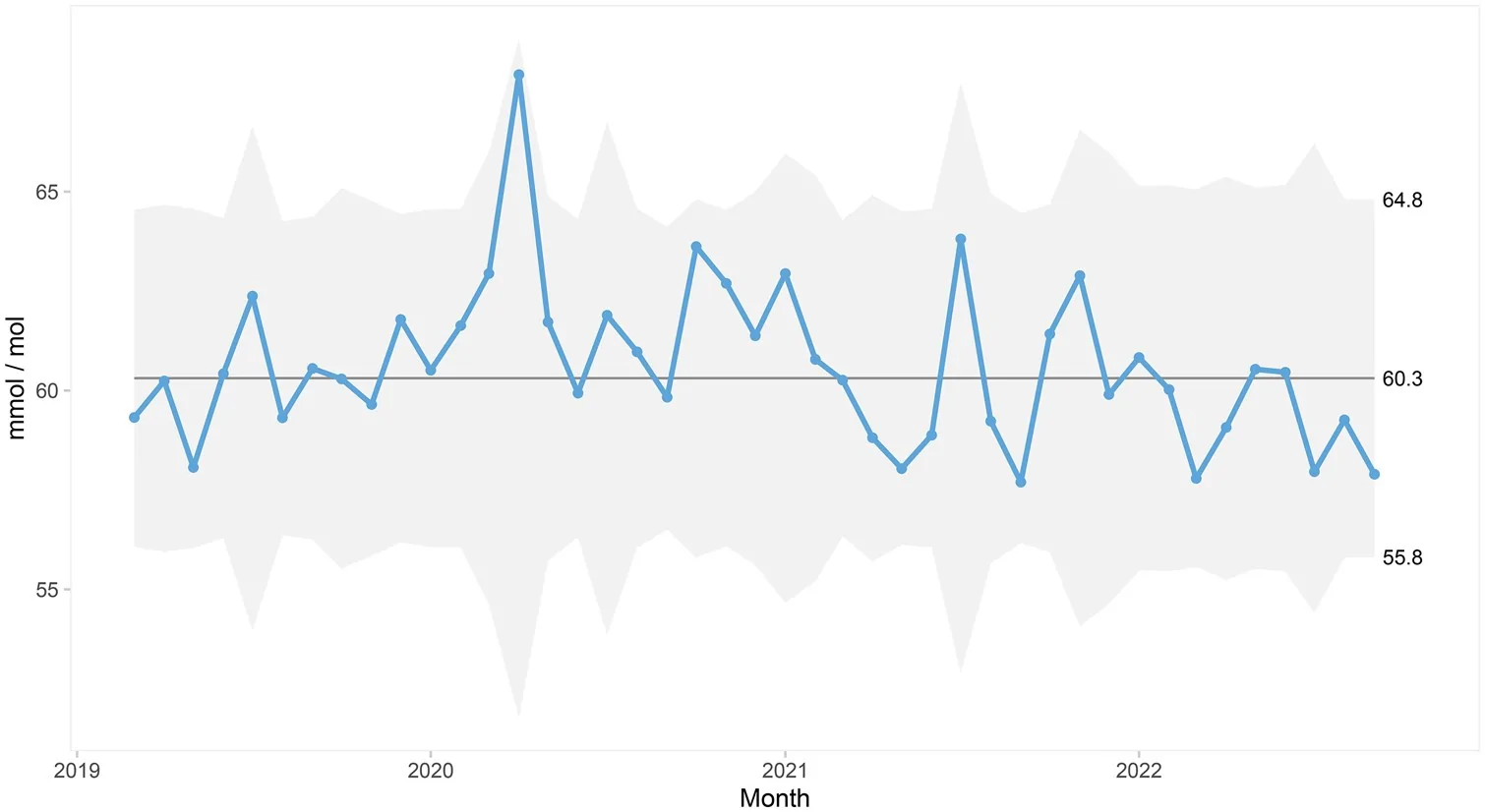
I′-chart of average HbA1c with denominator. Shaded area represents the area between the control limits.

So, when the subgroup size is taken into account, the apparent special cause in Fig. 1 falls within the limits of the expected common cause variation.

Also, notice that the centre lines are slightly different (60.6 vs 60.3), because the I′-chart uses the weighted rather than the unweighted mean of the averages.

### I′-chart for attribute data

In the following plots, the control limits from the I′-chart are superimposed as dashed lines on the original Shewhart charts.

Figure 3 is a P-chart of the percentage of patients with bacteremia who died within 30 days after infection. The I′-chart control limits are (in this case) a bit wider. The difference is, for practical purposes, negligible, which is generally the case when counts are low and follow the binomial distribution. However, this assumption frequently does not hold for higher counts, and the I′- and P′-charts are preferred.

**Figure 3:**
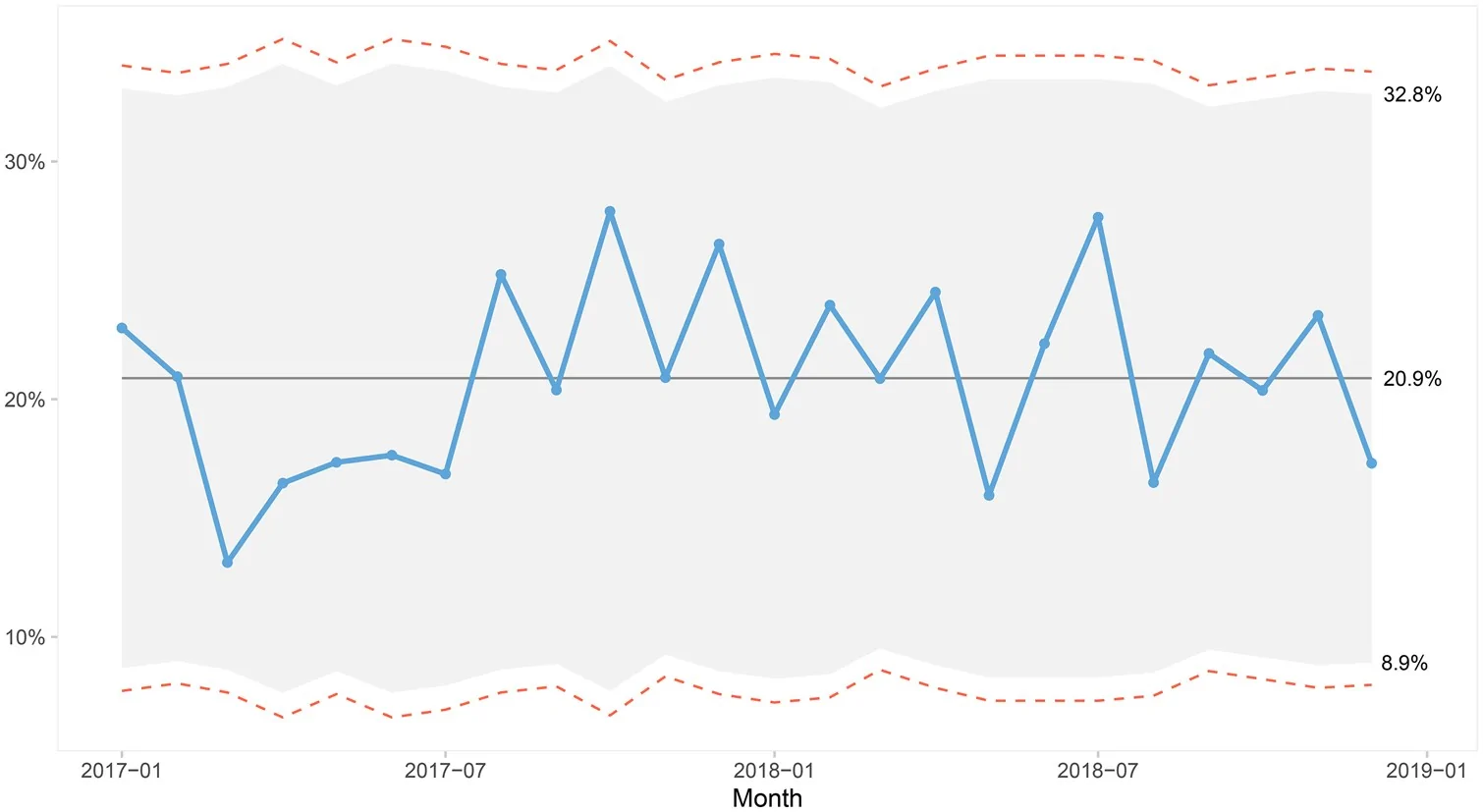
P-chart of bacteraemia mortality. Shaded area represents the area between the control limits; dashed lines represent the I′-chart limits.

Figure 4 plots the same data as Fig. 3, but the original control limits have been calculated using the procedure for P′-charts as suggested by Laney [16].

**Figure 4:**
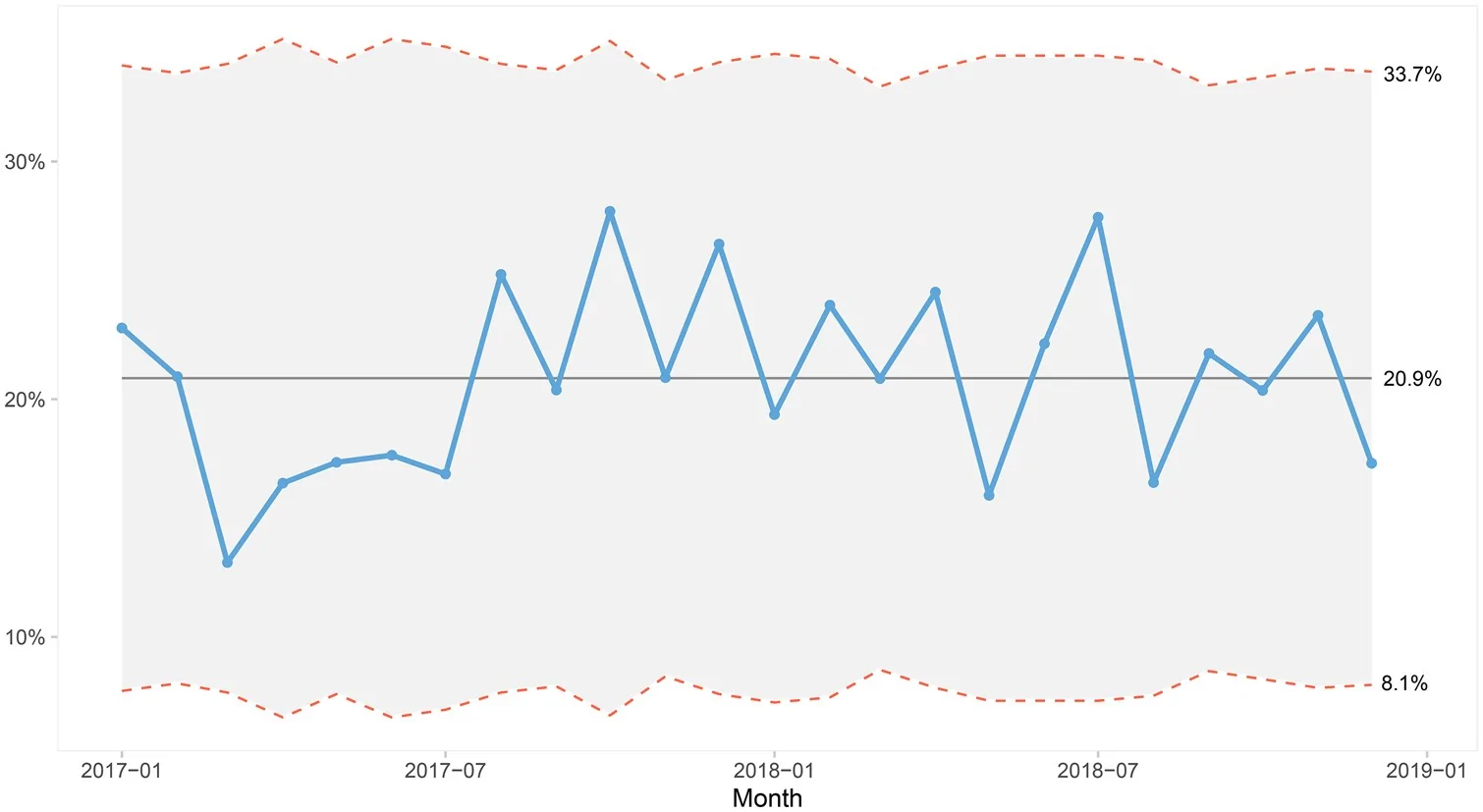
P′-chart of bacteraemia mortality. Shaded area represents the area between the control limits; dashed lines represent the I′-chart limits.

As seen (and expected from theory), the I′-chart limits match the P′-chart limits. The same applies to the U′-charts as demonstrated in Fig. 5.

**Figure 5:**
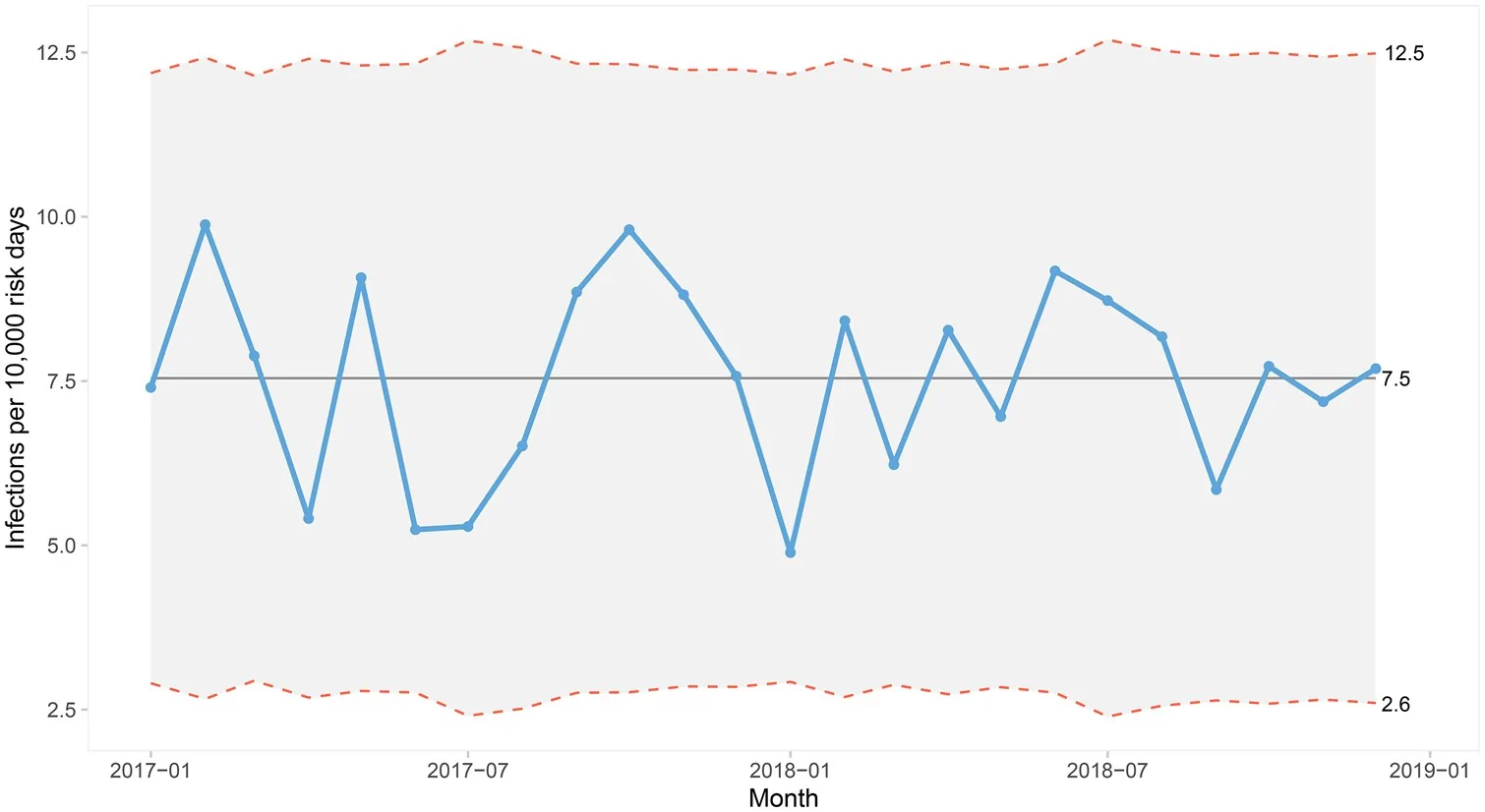
U′-chart of infection rates. Shaded area represents the area between the control limits; dashed lines represent the I′-chart limits.

Additional plots using simulated data are shown in the supplementary file. These plots are as follows.

Supplementary Figs 1 and 2 compare the I-chart and I′-chart for random data with subgroup size of one, and varying subgroup sizes from 80 to 120.

Supplementary Figs 3–5 compare the Xbar-chart and I′-chart for random data with varying subgroup sizes. Supplementary Figure 6 repeats this comparison for data containing a shift of 1 SD in the underlying mean after subgroup 12, to illustrate how the two charts behave in the presence of a special cause rather than under common-cause variation alone. The shift was evident as a sustained run above the centre line on both charts with comparable control limits.

Supplementary Figs 7 and 8 compare the P-chart and P′-chart with the I′-chart using random data with varying subgroup sizes.

Supplementary Figs 9–11 compare the C-chart, U-chart, and U′-chart with the I′-chart using random data with varying subgroup sizes.

In all charts that use simulated data, we find that the control limits from the I′-chart appear to be comparable with those from a traditional SPC chart and identical to those from the P′- and U′-charts.

## Discussion

### Statement of principal findings

We described and evaluated the I′-chart, a modified form of the traditional I-chart designed to accommodate varying subgroup sizes. Its development extends the familiar I-chart to a wider range of applications while retaining the simplicity, interpretability, and practicality, which make it attractive for routine use in healthcare improvement.

Our preliminary study identified three principal findings:

- When applied to data with constant subgroup sizes, the I′-chart produced results identical to those of the traditional I-chart, confirming that it is a generalization of the conventional I-chart.
- When subgroup sizes varied, the I′-chart adjusted the control limits according to subgroup size and estimated the process centre using a weighted rather than an unweighted average. By making differences in data precision explicit, the chart revealed, in one healthcare example, that an apparent special-cause signal was in fact consistent with common-cause variation once the reduced precision of that period’s data was accounted for.
- The control limits produced by the I′-chart using simulated data were broadly comparable with those of established SPC charts. For measurement data, the I′-chart produced limits comparable with the Xbar-S chart, while for attribute data it produced limits identical to those with Laney’s prime charts.

### Strengths and limitations

A strength of this preliminary study is that it combined simulated and real-world datasets. Simulated data allowed evaluation under controlled conditions and examination of the statistical properties of the proposed chart, while real-world datasets demonstrated its behaviour under the complexities encountered in healthcare practice, including non-normality, autocorrelation, seasonality and irregular sampling intervals. Together, these datasets provided an initial assessment of both the theoretical rationale and practical feasibility of the I′-chart.

Several limitations should be recognized. First, the evaluation compared the I′-chart primarily with the traditional I-chart and did not undertake a comprehensive comparison with alternative SPC methods across a broad range of healthcare applications. Second, the simulation studies focused mainly on stable processes for estimating sigma and control limits; only one special-cause scenario, a sustained shift of one SD in the mean, was examined (Fig. 2). Evaluation across a wider range of special-cause patterns, including trends and shifts of varying magnitude, together with supplementary signalling rules, is needed to establish the I′-chart’s sensitivity to special-cause variation. Third, the accuracy of control limits of the I′-chart can be affected if the moving range is distorted by outliers, where using the Nelson screening procedure (as in our examples) or the median moving range may help [10, 11].

Overall, this study provides an initial evaluation of the I′-chart and demonstrates its theoretical rationale and practical feasibility. However, further evaluation using both simulated and real-world data is required to establish the operating characteristics of the I′-chart. This should include a wider range of special-cause scenarios and signalling rules, comparisons across diverse healthcare applications, and assessment of its performance under the complexities encountered in routine practice.

### Interpretation within the context of the wider literature

The principal contribution of the I′-chart is not the introduction of an entirely new control chart, but the extension of the familiar I-chart to accommodate varying subgroup sizes. In doing so, it preserves the underlying philosophy of the traditional I-chart while broadening its applicability to subgroup summary data.

The origins of the I-chart have been described by Wheeler [16], who traced its development from Shewhart’s early work [16] on rational subgrouping in the 1920s. Shewhart recognized the value of analysing individual observations but lacked a practical method for estimating process variation. The subsequent introduction of successive differences and two-point moving ranges provided a simple empirical estimator of sigma [17] that led to the modern I-chart. This chart was popularized in the 1940s–50s but later fell into obscurity, although Levey and Jennings [18] successfully introduced it to laboratory medicine before it was reintroduced by Wheeler in the 1980s, who emphasized its value, based on his experience in the chemical industry, for monitoring one-at-a-time data provided the sampling was rational [9, 16].

Like the traditional I-chart, the I′-chart shares the general strengths and limitations of classical Shewhart charts [6]. Neither chart is well suited to continuous measurement data exhibiting pronounced seasonality, substantial autocorrelation, or severe departures from normality without appropriate preprocessing or alternative modelling. As with other Shewhart charts, both are relatively insensitive to small or gradual process shifts during ongoing monitoring, and the reliability of the estimated control limits is reduced when based on fewer than ∼20–25 observations. In addition, because both the I-chart and I′-chart estimate sigma from moving ranges, they are susceptible to distortion by extreme observations, for which robust alternatives, including the Nelson screening procedure and the median moving range, have been proposed [10].

The I-chart has been used since the 1940s [16, 19–21] and its performance has been examined by several authors [22–26] who have generally compared it with alternative SPC charts. By contrast, the present study evaluates an extension of the I-chart itself. In this respect, the I′-chart is conceptually similar to Laney’s prime charts [16] (P′, C′ and U′), which also extend established SPC methods to address recognized limitations in their underlying assumptions. However, the two extensions target different limitations. Laney’s prime charts retain the same theoretical σ as the traditional P- and U-chart, which already vary with subgroup size, but empirically correct that σ for over- or under-dispersion relative to the assumed binomial or Poisson model—addressing a distributional problem rather than a subgroup-size problem. The I′-chart, by contrast, retains the same empirical, distribution-free estimation of σ as the traditional I-chart, but corrects for the I-chart’s blindness to varying subgroup size. Unlike Laney’s prime charts, however, the I′-chart is applicable to both measurement and attribute data, providing a single approach for analysing subgroup summary data with varying subgroup sizes.

### Implications for policy, practice, and research

From a practical perspective, and subject to the limitations of this study, the I′-chart should replace the traditional I-chart whenever subgroup summary data with varying subgroup sizes are being analysed. Since the I′-chart is equivalent to the traditional I-chart when subgroup sizes are constant, but performs appropriately when they vary, routine use of the traditional I-chart in this setting is no longer justified.

More broadly, the I′-chart is best suited to settings where only subgroup summary data are available—a numerator and denominator per time point, rather than the individual-level observations underlying each subgroup—as is often the case with, for example, monthly infection counts and patient numbers. It also offers practical value where a single, consistent charting method is wanted across both measurement and attribute data, reducing the burden of selecting among Xbar-S, P-, C-, and U-charts.

Where individual-level observations are available, the Xbar-S chart remains the recommended SPC chart, because it estimates sigma directly from within-subgroup variation. The Xbar-S chart assumes that, under stable conditions, all subgroups are drawn from the same underlying process with a constant mean, so it attributes common-cause variation to a single source—variability within each subgroup—and estimates sigma accordingly. However, this assumption should not be taken for granted: as Janis [27] demonstrates, if subgroups carry fixed, systematic differences, then including that fixed bias in the estimate of sigma inflates the control limits and can mask genuine special-cause variation as ordinary common-cause noise. Where such fixed structural differences are plausible, practitioners should check for them (e.g. via boxplots by subgroup, or a formal decomposition of fixed and random variation) before relying on Xbar-S limits alone.

The I′-chart, by contrast, estimates sigma from the moving ranges between successive subgroup summaries. Because each summary already aggregates the observations within its subgroup, this moving-range estimate reflects both within-subgroup variation and between-subgroup variation, combined into a single source of information. Consequently, the two approaches are not designed to produce identical estimates of sigma and may therefore yield different control limits and signalling behaviour. Further methodological and empirical evaluation is required to establish the relative strengths and limitations of the two approaches and the circumstances in which each is most appropriate.

Indeed, rather than treating this difference as a limitation, we recommend that practitioners routinely run the I′-chart alongside the Xbar-S chart where both are available, since the two charts are sensitive to different sources of variation in the same underlying process: the Xbar-S chart isolates variation within subgroups, while the I′-chart reflects variation both within and between subgroups. Disagreement between the two is diagnostic—for example, a signal on one chart but not the other—may reflect the extent to which within- or between-subgroup variation is dominating the process, offering practitioners insight not available from either chart alone. This dual-charting recommendation follows a broader precedent in the SPC literature for comparing charts that estimate sigma from different sources. Mohammed and Worthington [28] demonstrated that plotting a traditional I-chart alongside a traditional attribute chart (P- or C-chart) can reveal an underlying systematic special cause that neither chart detects when used alone, since the two charts estimate sigma from fundamentally different sources. Taken together, these examples support a general principle: exclusive reliance on a single SPC chart risks missing sources of variation that only become visible when charts estimating sigma from different sources are compared side by side. The I′-chart and Xbar-S pairing extends this same principle to the specific question of subgroup-size-driven versus within-subgroup-driven variation.

One potential barrier to adoption is that the I′-chart requires more complex calculations than the traditional I-chart, because the control limits vary according to subgroup size. In practice, however, these calculations are readily automated within modern statistical software. We therefore recommend that SPC software incorporate the I′-chart as a standard chart type. This would facilitate wider evaluation in real-world settings while allowing users to compare the I′-chart directly with existing SPC methods, thereby improving understanding of the strengths and limitations of each approach.

Further methodological evaluation using both simulated and real-world data is required to establish the operating characteristics of the I′-chart. This should include assessment across a wider range of special-cause scenarios and signalling rules, comparisons across diverse healthcare applications, and evaluation of its performance under the complexities encountered in routine practice.

## Conclusion

The I′-chart has superior design properties to the traditional I-chart while preserving its simplicity and ease of interpretation. Our findings support replacing the traditional I-chart whenever subgroup summary data with varying subgroup sizes are analysed. Further methodological and empirical evaluation is required to establish its operating characteristics and determine its role alongside other SPC charts.

## Supplementary Material

mzag159_Supplementary_Data

## Data Availability

The datasets analysed in this study are provided in the supplementary files and a GitHub repository (https://github.com/anhoej/i_prime_chart) accompanying this article.
