# Supplementary material for: The I′-chart: a preliminary investigation of an improved individuals chart for quality improvement and control
Source: Int J Qual Health Care. 2026 Sep 26;38(4):mzag159. doi: 10.1093/intqhc/mzag159 (PMC13718905; doi:10.1093/intqhc/mzag159)
Supplement: Supplementary file 1 [file mzag159_supplementary_data.zip › 3_iprime_supp_code_20Sep2026.pdf]

The I'-chart: a preliminary investigation of an improved individuals chart for quality improvement and control

2026-09-20

SUPPLEMENTARY MATERIALS: R-CODE AND DATA SETS

R-code

```
################################################################################
# This R script constructs the figures for "Introducing the I'-chart:
# an improved individuals chart" by Anhøj, Taylor, and Mohammed.
#
# Data are provided as csv files: bacteremia.csv and diabetes_hba1c.csv.
#
# Plots are created by the qic() function from the qicharts2 package,
# version >= 0.8.0.
#
# 2026-09-20
################################################################################

# Load qicharts2 package ----

library(qicharts2)

# User defined function ----
# Function to plot control limits from the I'-chart on top of Shewhart chart
# x:     subgroup
# y:     numerator
# n:     denominator
# chart: Shewhart chart
# ...:   additional graphical parameters, e.g. titles

compplot <- function(x, y, n = NULL, chart, ...) {
  p1 <- qic(x, y, n, chart = chart, ...)  # original Shewhart chart
  p2 <- qic(x, y, n, chart = 'ip', ...)   # I'-chart

  p1 +                                       # original chart
    ggplot2::geom_line(ggplot2::aes(y = lcl),  # LCL from I'-chart
                       data   = p2$data,
                       linetype = 'dashed',
                       colour = 'tomato') +    # UCL from I'-chart
    ggplot2::geom_line(ggplot2::aes(y = ucl),
                       data   = p2$data,
                       linetype = 'dashed',
                       colour = 'tomato')
}

# Import data ----

## bacteremia data
bac   <- read.csv('bacteremia.csv',
                  comment.char = '#',
                  colClasses   = c('Date',
                                   'integer',
                                   'integer',
                                   'integer',
                                   'integer'))


## diabetes data
hba1c <- read.csv('diabetes_hba1c.csv',
                  comment.char = '#',
                  colClasses   = c('Date',
                                   'numeric',
                                   'integer'))

# Figures for main article ----

## Figure 1: I-chart of average HbA1c without denominator
qic(month, avg_hba1c,
```

```
    data = hba1c,
    chart = 'i',
    title = "Figure 1: I-chart of average HbA1c without denominator",
    ylab = 'mmol / mol',
    xlab = 'Month')

## Figure 2: I'-chart of average HbA1c with denominator
##     Multiply numerator by denominator to keep original scale
qic(month, avg_hba1c * n, n,
    data  = hba1c,
    chart = 'ip',
    title = "Figure 2: I'-chart of average HbA1c with denominator",
    ylab  = 'mmol / mol',
    xlab  = 'Month')

## Figure 3: P-chart of bacteremia mortality
compplot(bac$month, bac$deaths, bac$patients,
         chart = 'p',
         title = 'figure 3: P-chart of bacteremia mortality',
         xlab  = 'Month')

## Figure 4: P'-chart of bacteremia mortality
compplot(bac$month, bac$deaths, bac$patients,
         chart = 'pp',
         title = "Figure 4: P'-chart of bacteremia mortality",
         xlab  = 'Month')

## Figure 5: U'-chart of infection rates
compplot(bac$month, bac$ha_infections, bac$risk_days,
         chart    = 'up',
         multiply = 10000,
         title    = "Figure 5: U'-chart of infection rates",
         ylab     = 'Infections per 10,000 risk days',
         xlab     = 'Month')

# Supplementary plots ----

## Supplementary figure 1: I-chart, simulated normal data, no denominator
set.seed(1)                   # fixate random number generator
x <- 1:24                     # subgroup
y <- rnorm(24, 20, 3)         # numerator
n <- runif(24, 80, 120)       # denominator

compplot(x, y,
         chart = 'i',
         title = "Supplementary figure 1: I-chart, simulated normal data, no
denominator")

## Supplementary figure 2: I-chart, simulated normal data, varying denominators
compplot(x, y * n, n,
         chart = 'i',
         title = "Supplementary figure 2: I-chart, simulated normal data,
varying denominators")

## Supplementary figure 3: Xbar-chart, simulated normal data, seed 1
set.seed(1)
x <- rep(1:24, round(runif(24, 10, 20)))
y <- rnorm(length(x), 9)
compplot(x, y,
         chart = 'xbar',
         title = "Supplementary figure 3: Xbar-chart, simulated normal data,
seed 1")

## Supplementary figure 4: Xbar-chart, simulated normal data, seed 6
```

```
set.seed(6)
x <- rep(1:24, round(runif(24, 10, 20)))
y <- rnorm(length(x), 9)
compplot(x, y,
         chart = 'xbar',
         title = "Supplementary figure 4: Xbar-chart, simulated normal data,
seed 6")

## Supplementary figure 5: Xbar-chart, simulated normal data, seed 8
set.seed(8)
x <- rep(1:24, round(runif(24, 10, 20)))
y <- rnorm(length(x), 9)
compplot(x, y,
         chart = 'xbar',
         title = "Supplementary figure 5: Xbar-chart, simulated normal data,
seed 8")

## Supplementary figure 6: Xbar-chart, simulated normal data, mean shift
set.seed(8)
x <- rep(1:24, round(runif(24, 10, 20)))
y[x > 12] <- rnorm(length(x[x > 12]), 10)
compplot(x, y,
         chart = 'xbar',
         title = "Supplementary figure 6: Xbar-chart, simulated normal data,
mean shift")

## Supplementary figure 7: P-chart, simulated binomial data
set.seed(1)
x <- 1:24
n <- round(runif(24, 100, 120))
y <- rbinom(24, n, 0.1)
compplot(x, y, n,
         chart = 'p',
         title = "Supplementary figure 7: P-chart, simulated binomial data")

## Supplementary figure 8: P'-chart, simulated binomial data
compplot(x, y, n,
         chart = 'pp',
         title = "Supplementary figure 8: P'-chart, simulated binomial data")

## Supplementary figure 9: C-chart, simulated Poisson data
set.seed(2)
x <- 1:24
n <- (runif(24, 90, 110))
y <- rpois(24, 25)
compplot(x, y,
         chart = 'c',
         title = "Supplementary figure 9: C-chart, simulated Poisson data")

## Supplementary figure 10: U-chart, simulated Poisson data
compplot(x, y, n,
         chart = 'u',
         title = "Supplementary figure 10: U-chart, simulated Poisson data")

## Supplementary figure 11: U'-chart, simulated Poisson data
compplot(x, y, n,
         chart = 'up',
         title = "Supplementary figure 11: U'-chart, simulated Poisson data")
```

Bacteremia data

bacteremia.csv

```
# Bacteremia
#
# Hospital acquired and all cause bacteremias and 30 days mortality
#
# Variables:
#   month (date): month of infection
#   ha_infections (numeric): number of hospital acquired infections
#   risk_days (numeric): number of patient days without infection
#   deaths (numeric): 30-day mortality after all-cause infection
#   patients (numeric): number of patients with all-cause infection

month,ha_infections,risk_days,deaths,patients
2017-01-01,24,32421,23,100
2017-02-01,29,29349,22,105
2017-03-01,26,32981,13,99
2017-04-01,16,29588,14,85
2017-05-01,28,30856,17,98
2017-06-01,16,30544,15,85
2017-07-01,14,26482,15,89
2017-08-01,18,27637,25,99
2017-09-01,27,30495,21,103
2017-10-01,30,30600,24,86
2017-11-01,28,31770,23,110
2017-12-01,24,31679,26,98
2018-01-01,16,32720,18,93
2018-02-01,25,29698,23,96
2018-03-01,20,32118,24,115
2018-04-01,25,30212,25,102
2018-05-01,22,31612,15,94
2018-06-01,28,30514,21,94
2018-07-01,23,26362,26,94
2018-08-01,23,28131,16,97
2018-09-01,17,29071,25,114
2018-10-01,22,28481,22,108
2018-11-01,21,29223,24,102
2018-12-01,22,28611,18,104
```

Diabetes data

diabetes_hba1c.csv

```
# Diabetes HbA1c
#
# HbA1c measurements in children with diabetes
#
# Variables:
#   month (date): month of measurements
#   avg_hba1c (numeric): average of HbA1c measurements
#   n (integer): number of patients who visited the clinic

month,avg_hba1c,n
2019-03-01,59.3224299065421,214
2019-04-01,60.2413793103448,203
2019-05-01,58.0660377358491,212
2019-06-01,60.4243697478992,238
2019-07-01,62.375,96
2019-08-01,59.3145161290323,248
2019-09-01,60.5555555555556,234
2019-10-01,60.2916666666667,168
2019-11-01,59.64948453608249,194
2019-12-01,61.787610619469,226
2020-01-01,60.509345794392495,214
2020-02-01,61.6338028169014,213
2020-03-01,62.9411764705882,119
2020-04-01,67.9433962264151,53
2020-05-01,61.7228260869565,184
2020-06-01,59.9336099585062,241
2020-07-01,61.8924731182796,93
2020-08-01,60.9718309859155,213
2020-09-01,59.8308270676692,266
2020-10-01,63.6210526315789,190
2020-11-01,62.6944444444444,216
2020-12-01,61.3771428571429,175
2021-01-01,62.9421487603306,121
2021-02-01,60.7837837837838,148
2021-03-01,60.2622950819672,244
2021-04-01,58.8126373626374,182
2021-05-01,58.0365296803653,219
2021-06-01,58.8779342723005,213
2021-07-01,63.8114285714286,70
2021-08-01,59.2290502793296,179
2021-09-01,57.6964285714286,224
2021-10-01,61.4228855721393,201
2021-11-01,62.8888888888889,99
2021-12-01,59.9,120
2022-01-01,60.8309090909091,165
2022-02-01,60.0243902439024,164
2022-03-01,57.78947368421049,171
2022-04-01,59.073333333333295,150
2022-05-01,60.5357142857143,168
2022-06-01,60.4601226993865,163
2022-07-01,57.9603603603604,111
2022-08-01,59.26315789473681,190
2022-09-01,57.895287958115205,191
```
