# Supplementary material for: The I′-chart: a preliminary investigation of an improved individuals chart for quality improvement and control
Source: Int J Qual Health Care. 2026 Sep 26;38(4):mzag159. doi: 10.1093/intqhc/mzag159 (PMC13718905; doi:10.1093/intqhc/mzag159)
Supplement: Supplementary file 1 [file mzag159_supplementary_data.zip › 4_iprime_supp_figs_20Sep2026.pdf]

The I'-chart: a preliminary investigation of an improved individuals chart for quality improvement and control

SUPPLEMENTARY FIGURES

I-charts from individual random normal measurements

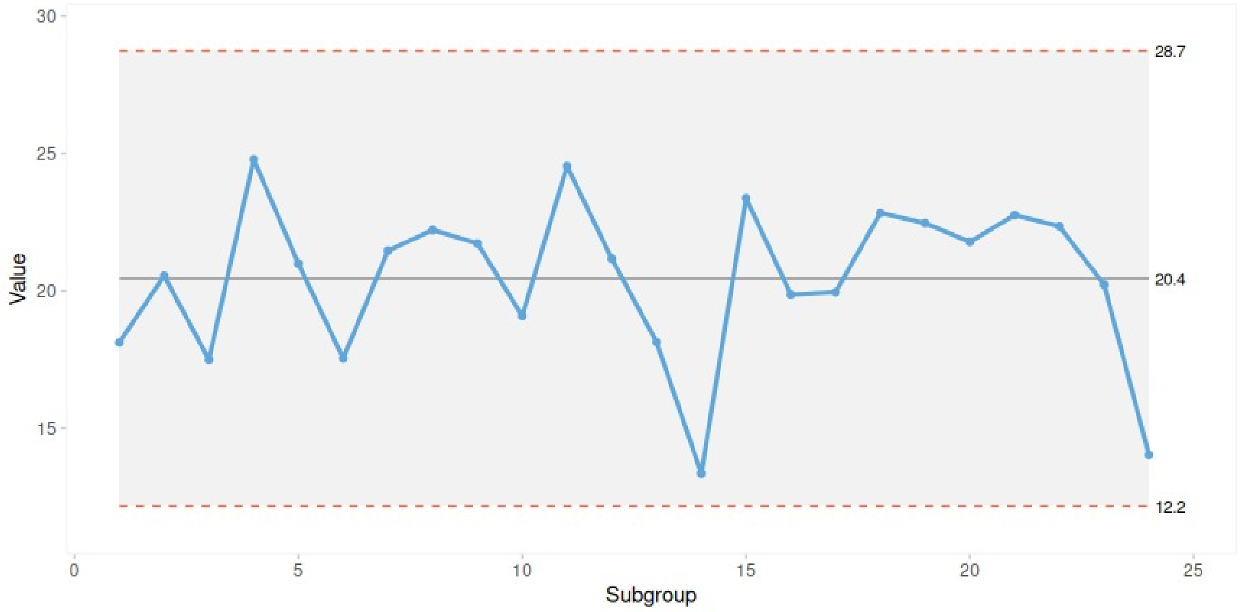

Supplementary figure 1: ***I-chart, simulated normal data, no denominator*** *(shaded area represents the area between the control limits, dashed lines represent the I'-chart limits). [ALT TEXT: I-chart of 24 simulated normal data points (mean 20, SD 3) with I-chart control limits shown as shaded background and I'-chart limits as dashed lines, the two overlapping exactly since subgroup size is constant.]*

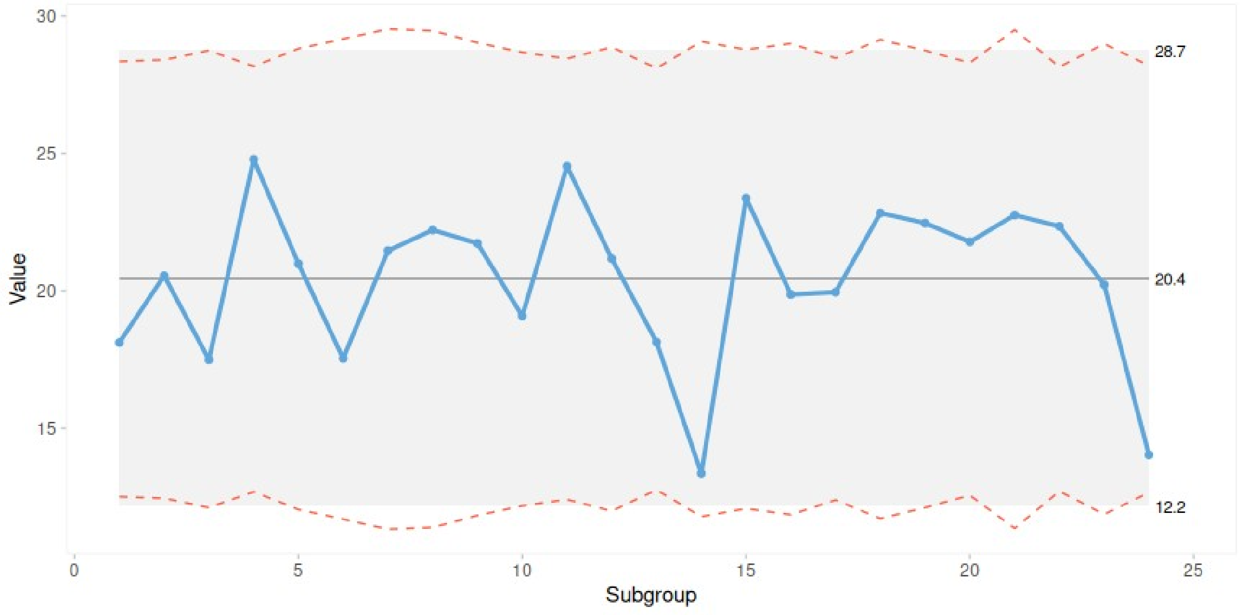

Supplementary figure 2: ***I-chart, simulated normal data, varying denominators (80–120)*** *(shaded area represents the area between the control limits, dashed lines represent the I′-chart limits). [ALT TEXT: I′-chart of the same simulated data as Figure 6 but with subgroup sizes ranging from 80 to 120, showing I′-chart control limits that vary in width compared with the constant I-chart limits.]*

Xbar-charts from multiple random normal measurements

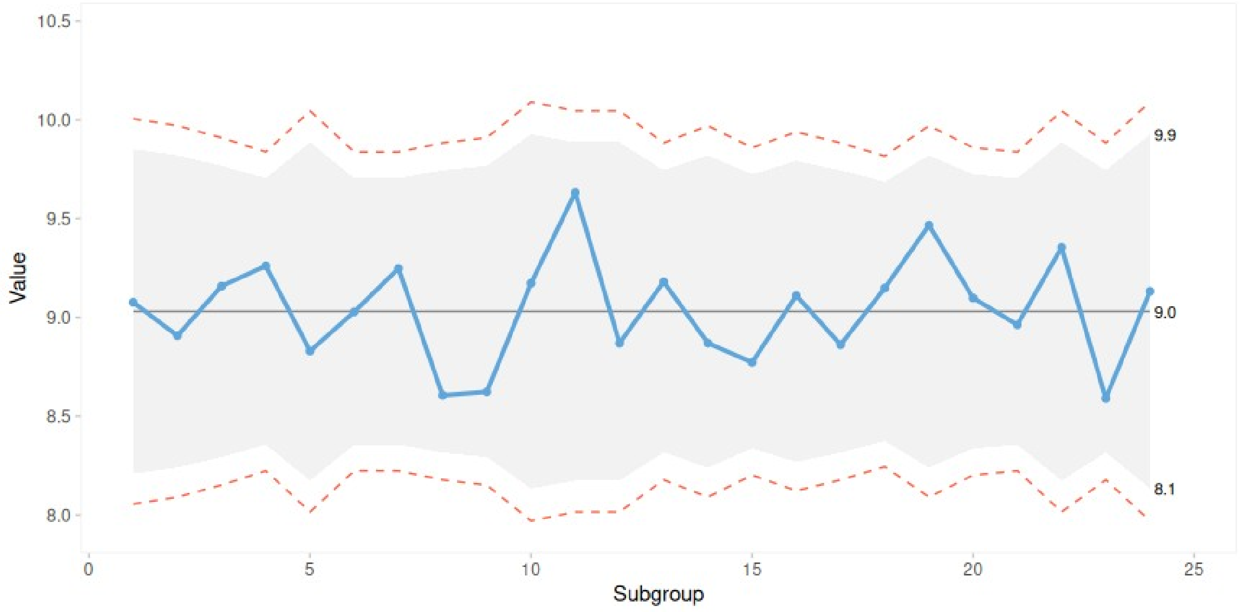

Supplementary figure 3: ***Xbar-chart, simulated normal data, seed 1*** *(shaded area represents the area between the control limits, dashed lines represent the I′-chart*

limits). [ALT TEXT: Xbar-chart of 24 subgroup means (mean 9, SD 1) with shaded Xbar-chart control limits and dashed I′-chart limits, the I′-chart limits slightly wider.]

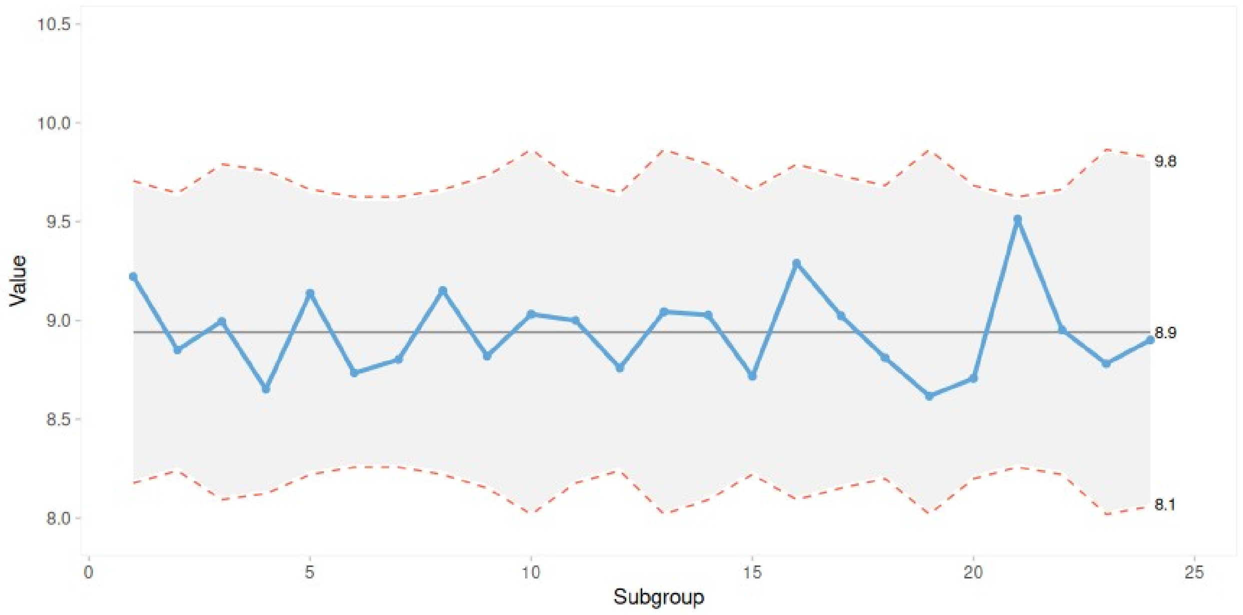

*Supplementary figure 4: **Xbar-chart, simulated normal data, seed 6** (shaded area represents the area between the control limits, dashed lines represent the I′-chart limits). [ALT TEXT: Xbar-chart of 24 subgroup means (mean 9, SD 1, different random seed) with shaded Xbar-chart control limits and dashed I′-chart limits that are close to identical.]*

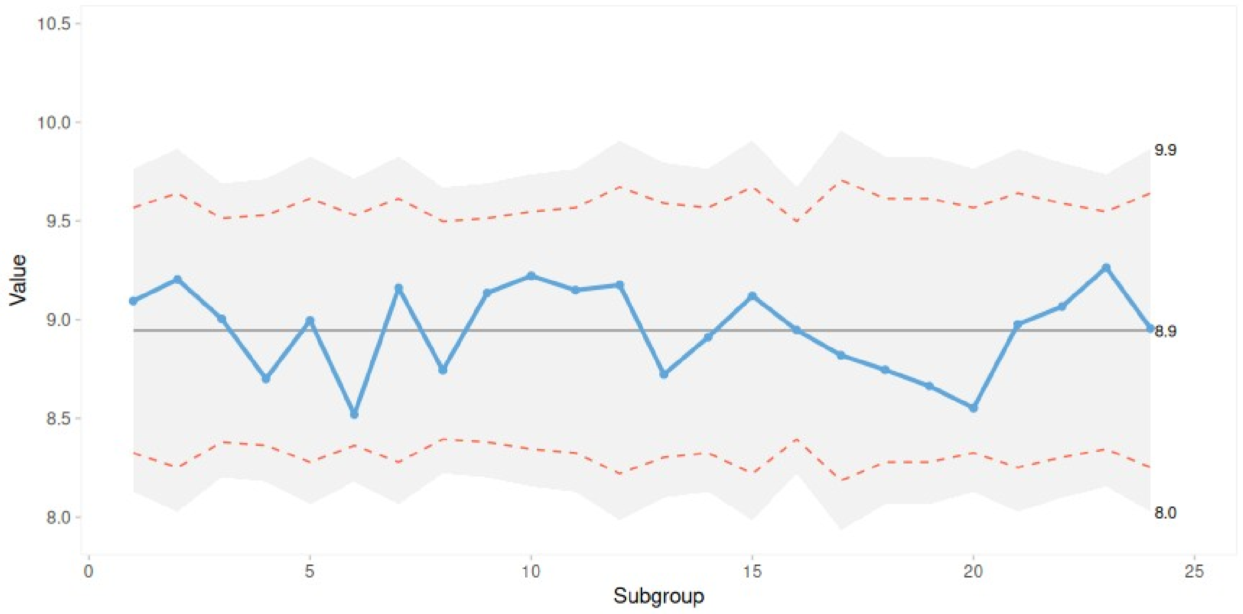

*Supplementary figure 5: **Xbar-chart, simulated normal data, seed 8** (shaded area represents the area between the control limits, dashed lines represent the I′-chart*

limits). [ALT TEXT: Xbar-chart of 24 subgroup means (mean 9, SD 1, different random seed) with shaded Xbar-chart control limits and dashed I′-chart limits that are slightly tighter.]

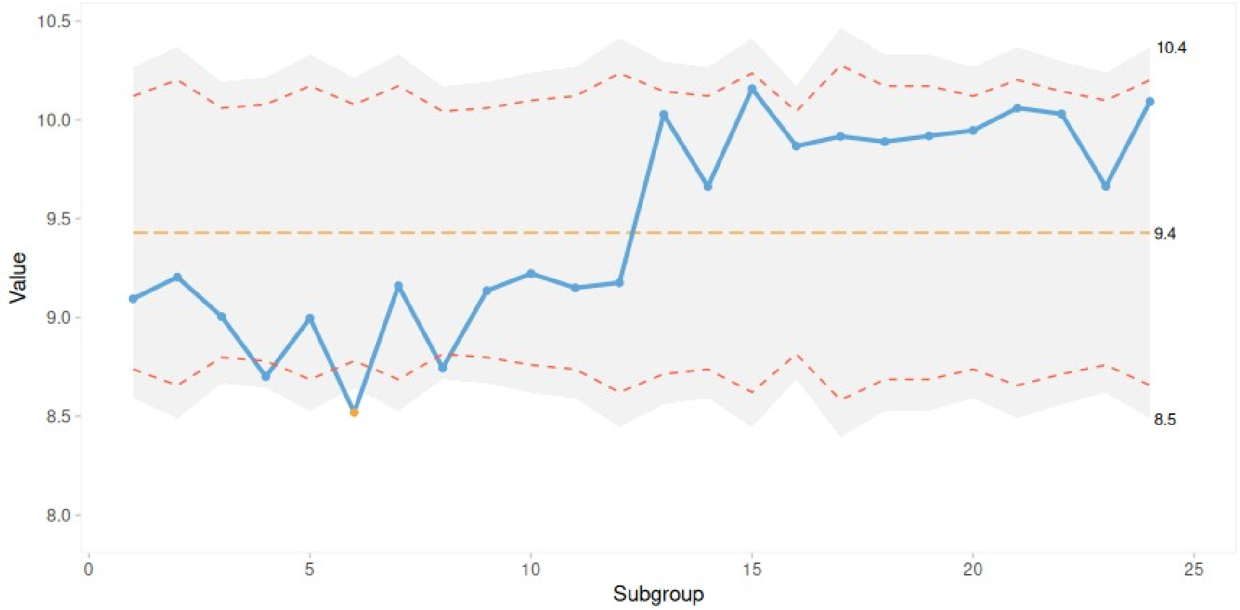

Supplementary figure 6: ***Xbar-chart, simulated normal data, mean shift*** *(shaded area represents the area between the control limits, dashed lines represent the I′-chart limits). [ALT TEXT: Xbar-chart of 24 subgroup means with a 1 SD upward shift in the mean introduced after subgroup 12, shown as a step change in the centre line; shift is signalled by a data point (#6) below the lower control limits and two long runs of data point below (#1-12) and above (#13-24) the centre line.]*

P- and P′-charts from random binomial data

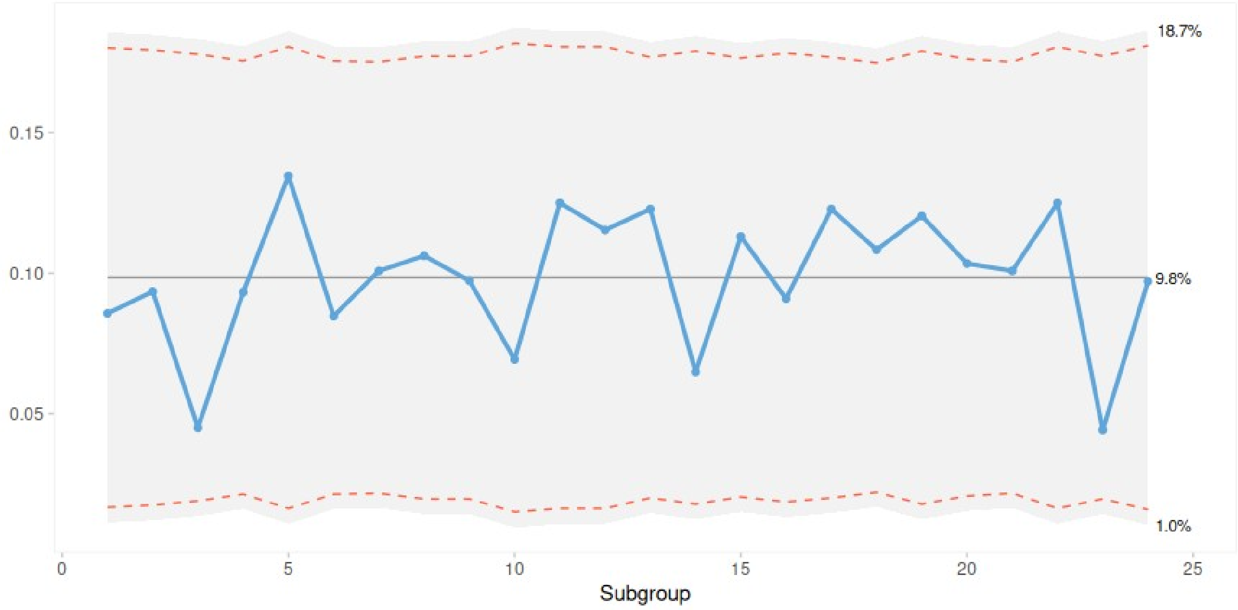

Supplementary figure 7: ***P-chart, simulated binomial data*** *(shaded area represents the area between the control limits, dashed lines represent the I′-chart limits). [ALT TEXT: P-chart of 24 simulated proportions from a binomial distribution (p = 0.1) with shaded P-chart control limits and overlaid dashed I′-chart limits that are slightly wider.]*

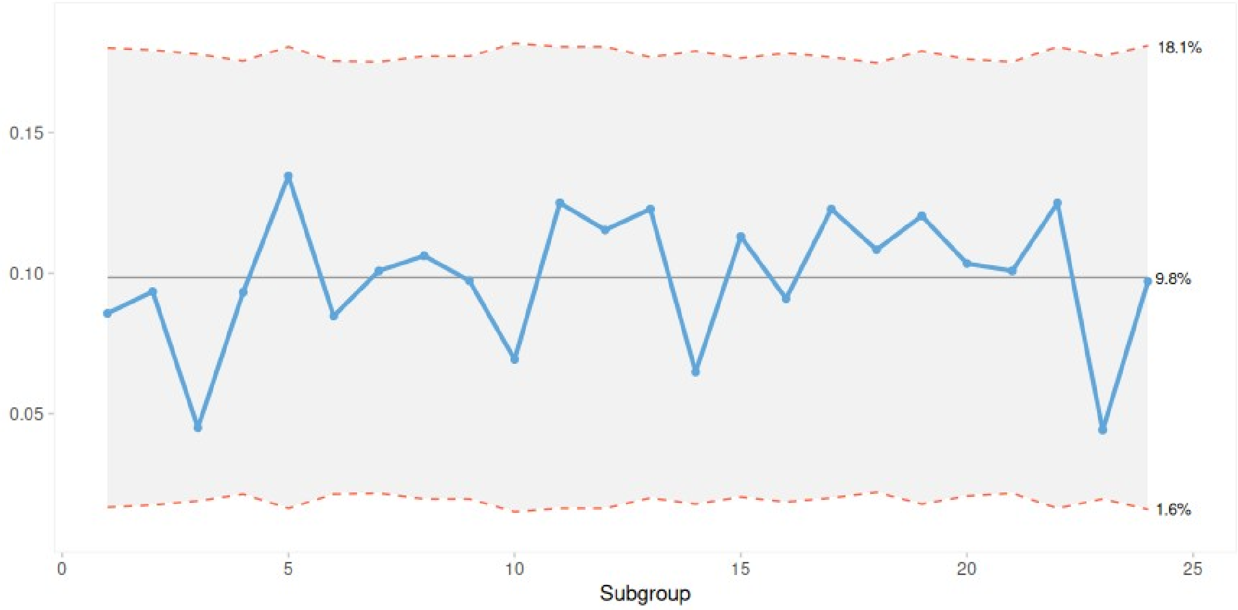

Supplementary figure 8: ***P′-chart, simulated binomial data*** *(shaded area represents the area between the control limits, dashed lines represent the I′-chart limits). [ALT TEXT: Same binomial simulation as Figure 12 with shaded P′-chart control limits and overlaid dashed I′-chart limits that closely match.]*

C-, U- and U’-charts from random Poisson data

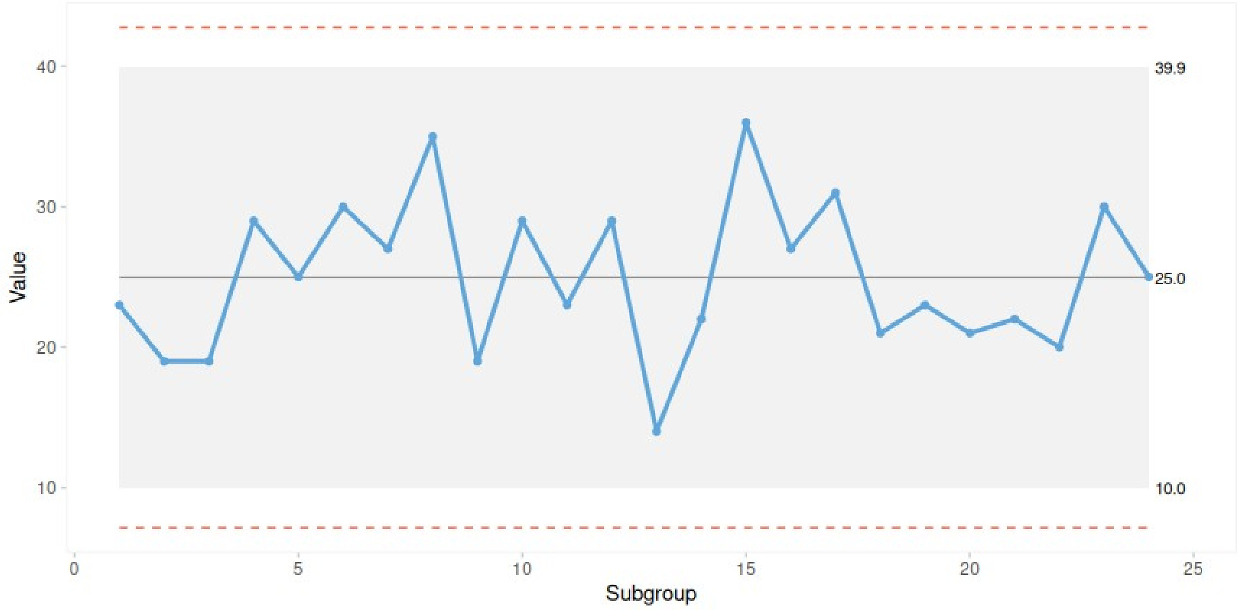

Supplementary figure 9: ***C-chart, simulated Poisson data*** *(shaded area represents the area between the control limits, dashed lines represent the I’-chart limits). C-chart of 24 simulated counts from a Poisson distribution (mean 25) with shaded C-chart control limits and overlaid dashed I’-chart limits that are noticeably wider.*

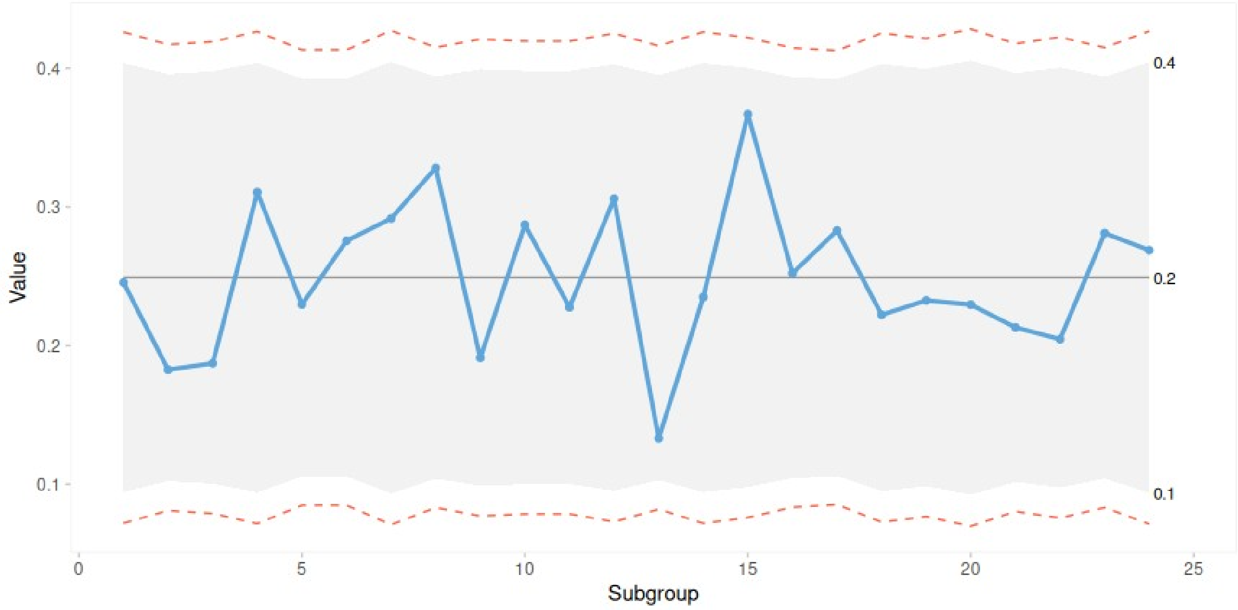

Supplementary figure 10: ***U-chart, simulated Poisson data*** *(shaded area represents the area between the control limits, dashed lines represent the I’-chart limits). [ALT TEXT: U-chart of 24 simulated rates from a Poisson distribution (mean 25) with varying*

subgroup sizes (90-110), shaded U-chart control limits, and overlaid dashed I′-chart limits that are wider.]

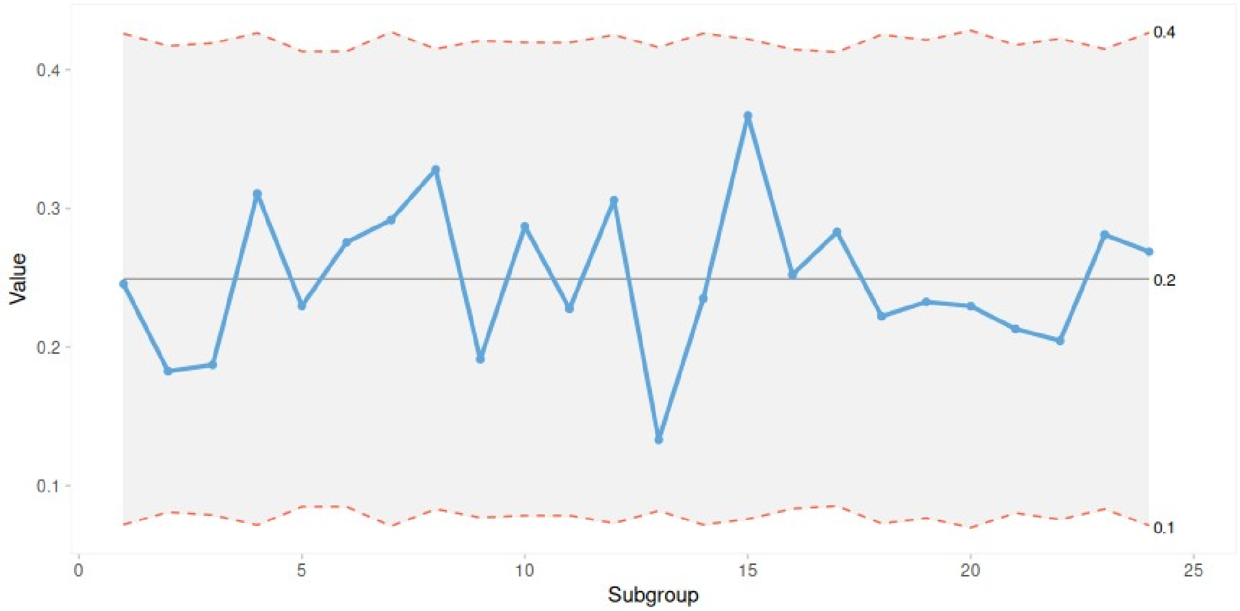

Supplementary figure 11: ***U′-chart, simulated Poisson data*** *(shaded area represents the area between the control limits, dashed lines represent the I′-chart limits). [ALT TEXT: Same Poisson rate simulation as Figure 15 with shaded U′-chart control limits and overlaid dashed I′-chart limits that closely match.]*
